# The correlation between nutrition and frailty and the receiver operating characteristic curve of different nutritional indexes for frailty

**DOI:** 10.1186/s12877-021-02580-5

**Published:** 2021-11-02

**Authors:** Hong Liang, Xiaoping Li, Xiaoye Lin, Yanmin Ju, Jiyan Leng

**Affiliations:** 1 Department of Cadre Ward, The First Hospital of Jilin University, Changchun, China; 2 Department of Pediatrics, The First Hospital of Jilin University, Changchun, China

**Keywords:** Frailty, Nutrition, Inpatients

## Abstract

**Background:**

Frailty is a kind of geriatric syndrome, which is very common in the elderly. Patients with malnutrition are at higher risk of frailty. This study explored the correlation between nutrition and frailty and compared the receiver operating characteristic curve of different nutritional indexes for frailty.

**Methods:**

This cross-sectional study included 179 inpatients aged ≥65 years old. Frailty was measured using Fried Frailty Phenotype, handgrip strength was measured using JAMAR@Plus and the 4.57 m usual gait speed was measured using a stopwatch. Comprehensive nutritional assessment refers to the application of Mini Nutritional Assessment (MNA) to assess the nutritional status of patients.

**Results:**

Compared with the non-frailty group, the upper arm circumference, calf circumference, hemoglobin, albumin, prealbumin, cholesterol and low density lipoprotein in the frailty group were lower (*P* < 0.05). Comprehensive nutritional assessment, whether as a categorical variable or a continuous variable, was significantly correlated with frailty (*P* < 0.05). Model1 showed that the risk of frailty in malnourished patients was 3.381 times higher than that in well nourished patients (*P* = 0.036). Model2 showed that the risk of frailty decreased by 13.8% for every 1 point increase in MNA score (*P* = 0.009). The area under the curves of albumin, prealbumin and hemoglobin was larger (AUC > 0.65), AUC was 0.718, 0.693 and 0.743, respectively.

**Conclusions:**

Our results suggest that malnutrition is closely related to frailty. As for single nutritional indexes, albumin, prealbumin and hemoglobin were found to be associated with frailty. Further cohort studies are needed to verify their ability to screen for frailty.

## Introduction

Frailty is undoubtedly one of the most serious global public health challenges we will confront in the coming century. The rapid expansion of the aging population has led to a corresponding increase in the number of frail elderly [1, 2]. Frailty is defined as a clinical state in which individuals are more likely to have health-related negative events when exposed to endogenous or exogenous stressors [3, 4]. Frailty is a dynamic evolution process from health to weakness, in which the degree of weakness can develop to any direction with the passage of time [2]. Accordingly, frailty may be reversible, and its related functional decline is also a disease that may be prevented [5].

Nutrition is a crucial factor closely related to frailty syndrome [6]. Malnutrition can significantly aggravate the development of frailty, because long-term malnutrition, insufficient protein and energy intake can lead to weight loss (one of the five criteria of frailty phenotype), fatigue, weakness, slow walking speed and low level of physical activity [7]. The pathophysiology of frailty is complex and multifactorial, and nutrition is an important mechanism of its pathogenesis and a specific target of treatment [8]. People are more and more interested in the relationship between frailty and nutrition, but previous studies rarely compared the relationship between different nutritional indicators and frailty, so these variables were studied in this study. The results can be used as an important clue to provide nutritional care for elderly patients.

## Materials and methods

### Study design

This work is a cross-sectional study conducted in the cadre ward of the First Hospital of Jilin University from December 2020 to June 2021. In patients aged 65 years or above were recruited. Patients who were unable to walk for neuromusculoskeletal diseases such as Parkinson’s disease, severe knee or hip osteoarthritis, lumbar spinal stenosis, and Participants with malignant tumors, severe cognitive impairment, autoimmune diseases, severe liver and kidney dysfunction were excluded from the study. The study was in line with the declaration of Helsinki Declaration and approved by the ethics committee of the First Hospital of Jilin University.

### Measurements

#### General information

Age, gender and exercise were evaluated by self-made face-to-face interview questionnaire.

Clinical information comes from electronic medical records, including smoking history, drinking history, types and diseases of chronic diseases, hemoglobin, albumin, prealbumin, cholesterol, triglyceride and low density lipoprotein Cholesterol.

#### Anthropometric measurements

1. Height and weight were measured according to clinical standards.
2. Body mass index (BMI) was calculated as weight (kg) divided by height (m) squared.
3. Walking speed was calculated as 4.57 m walk time divided by 4.57 m [9, 10]. Participants walked at daily speed. Time was recorded with a stopwatch (accurate to 0.01 s). The average value was calculated after the second measurement.
4. Handgrip strength of the dominant hand was measured by handgrip dynamometer (JAMAR@Plus) for 3 times, and the maximum handgrip strength was taken for analysis [11, 12].
5. Participants stood with their feet shoulder width apart and the calf circumference around the most prominent part of the gastrocnemius muscle was measured (accurate to 0.1 cm).
6. Arm circumference at the level of the thickest part of biceps brachii muscle was measured with the upper limb sags naturally (accurate to 0.1 cm).

#### Frailty assessment

Frailty was defined as a major biological phenotype by Fried Frailty Phenotype [13]. Satisfying three or more of the five followings were diagnosed as frailty syndrome, including weight loss of more than 4.5 kg or > 5%, fatigue, less physical activity, slow pace and weak handgrip in the past year. Fatigue refers to feeling that everything needs effort or can’t move forward for three or more days. The decrease of handgrip strength was estimated by lower limit of handgrip strength which was determined according to the participants’ gender and body mass index. Decreased handgrip strength of male: BMI ≤ 24.0 kg/m^2^, handgrip strength ≤29 kg; 24.1 ≤ BMI ≤ 28.0 kg/m^2^, handgrip strength ≤30 kg; BMI > 28 kg/m^2^, handgrip strength ≤32 kg. Decreased handgrip strength of female: BMI ≤ 23.0 kg/m^2^, handgrip strength ≤17 kg; 23.1 ≤ BMI ≤ 26.0 kg/m^2^, handgrip strength ≤17.3 kg; 26.1 ≤ BMI ≤ 29.0 kg/m^2^, handgrip strength ≤18 kg; BMI > 29 kg/m^2^, handgrip strength ≤21 kg. Male < 383 kcal / week or female < 270 kcal / week was judged as physical activity decrease. Walking speed was calculated by the time required to walk 4.57 m, male with height ≤ 173 cm, walking speed ≥7 s or height > 173 cm, walking speed ≥6 s was identified as walking speed descent; while the standard of walking speed descent among female is height ≤ 159 cm, walking speed ≥7 s or height > 159 cm, walking speed ≥6 s.

#### Malnutrition

Mini Nutritional Assessment (MNA) is a nutritional assessment tool with 18 questions specially developed for the elderly. It consists of four parts: anthropometry (body mass index, calf circumference and arm circumference measurement), self-reported health status, dietary problems (including weight loss) and clinical health status. MNA was used to comprehensively evaluate the nutritional status of elderly patients [14, 15]. The total score of MNA is 30 points, if the score was < 17 points, patients were classified as malnutrition; if the score was between 17 to 23.5 points, patients were at the risk of malnutrition; good nutrition, if the score was > 23.5 points.

### Data analysis

SPSS/WIN 23.0 software (IBM Corp., Armonk, NY, USA) or MedCalc 18.2.1 (MedCalc, Mariakerke, Belgium) was used for statistical analysis. Kolmogorov-Smirnov test was used to test the normality of continuous variables. Continuous variables were presented as mean ± standard deviation or median (IQR) based on the distribution characteristics of the data. Student’s t-test or Mann-Whitney U test was used to test the difference between two groups. Classified variables were described as absolute number and percentages, and the difference between two groups was test by Chi-squared test. Multivariable logistic regression model was used to explore the association between malnutrition and frailty. The AUC value was calculated by drawing ROC curve to identify the association between different nutritional indexes and senile frailty. The Youden index was used to determine the optimal cutoff point. Comparison of two ROC curves was based on the method of Delong et al. [16]. Values of *p* < 0.05 were considered statistically significant.

## Results

### General baseline characteristics of patients

As shown in Table 1, a total of 179 participants participated in the study. There were 60 patients in the frailty group with an average age of 84.87 ± 7.79, and 119 patients in the non-frailty group with an average age of 77.37 ± 8.98. The age difference between the two groups was statistically significant (*P* < 0.001). In addition, there were significant differences in the proportion of smoking and exercise. The difference in the number of chronic diseases, activities of daily living score, MNA score and nutritional status between the two groups were also exhibit statistically significant (*P* < 0.05).

**Table 1:** General baseline characteristics

Variates	Frailty (*n* = 60)	Non- Frailty (*n* = 119)	*P*
Age, mean (SD), years	84.87 ± 7.79	77.37 ± 8.98	< 0.001
Sex, male, n (%)	42 (70.0)	96 (80.7)	0.109
Smoke, n (%)	33 (55.0)	45 (37.8)	0.029
Drink, n (%)	28 (46.7)	47 (39.5)	0.359
Exercise, n (%)	17 (28.3)	89 (74.8)	< 0.001
Number of chronic diseases, median (IQR)	3 (2,3)	2 (2,3)	0.017
ADL, median (IQR)	30.00 (23.25,40.00)	16.00 (14.00,22.00)	< 0.001
MNA, median (IQR)	16.75 (15.50,22.00)	23.50 (21.00,26.00)	< 0.001
MNA Classification, n (%)			< 0.001
Well-nourished	9 (15.0)	51 (42.8)	
At risk of malnutrition	21 (35.0)	49 (41.2)	
Malnourished	30 (50.0)	19 (16.0)	

### Comparison of the anthropometric parameters and the nutrition index of laboratory between the frailty group and the non-frailty group

As shown in Table 2, compared with the non-frailty group, the upper arm circumference, calf circumference, hemoglobin, albumin, prealbumin, cholesterol and low density lipoprotein of patients in the frailty group were lower (*P* < 0.05).

**Table 2:** Comparison of nutritional indexes between the frailty group and the non- frailty group

Variates	Frailty (*n* = 60)	Non- Frailty (*n* = 119)	*P*
BMI, median (IQR), kg/m^2^	23.80 (21.85,25.40)	24.20 (22.60,26.10)	0.103
AC, median (IQR), cm	25.80 (24.35,26.95)	26.60 (25.40,28.00)	0.008
CC, mean (SD), cm	30.92 ± 3.47	32.61 ± 3.33	0.002
Hb, mean (SD), g/L	122.35 ± 18.76	139.92 ± 18.05	< 0.001
Alb, mean (SD), g/L	35.83 ± 3.59	39.27 ± 4.59	< 0.001
Prealbumin, mean (SD), g/L	0.19 ± 0.06	0.24 ± 0.06	< 0.001
Cholesterol, mean (SD), mmol/L	4.12 ± 1.12	4.57 ± 1.09	0.012
Triglyceride, median (IQR), mmol/L	1.11 (0.81,1.62)	1.31 (0.88,1.77)	0.150
LDL-C, mean (SD), mmol/L	2.73 ± 0.92	3.07 ± 0.86	0.016

### Relationship between comprehensive nutritional assessment and frailty

As shown in Table 3, we incorporated the comprehensive assessment of nutrition into the Logistic regression model in the form of categorical variables and continuous variables to explore the relationship between nutrition and frailty. The results showed that nutrition was significantly correlated with weakness, whether as a categorical variable or a continuous variable (*P* < 0.05). Model1 showed that the risk of frailty in malnourished patients was 3.381 times higher than well-nourished patients (*P* = 0.036). Model2 showed that the risk of frailty decreased by 13.8% for every 1 point increase in MNA score (*P* = 0.009).

**Table 3:** Comprehensive assessment of nutrition and frailty

Variates	OR (95% CI)	*P*
Model 1		
Age	1.043 (0.984–1.106)	0.154
Smoke	0.983 (0.415–2.326)	0.968
Exercise	0.327 (0.136–0.787)	0.013
Number of chronic diseases	0.419 (1.174–1.733)	0.419
ADL	1.106 (1.041–1.174)	0.001
MNA Classification		
Well-nourished	1	–
At risk of malnutrition	1.405 (0.477–4.138)	0.537
Malnourished	3.381 (1.086–10.528)	0.036
Model 2		
Age	1.046 (0.986–1.109)	0.140
Smoke	1.016 (0.428–2.410)	0.972
Exercise	0.338 (0.140–0.816)	0.016
Number of chronic diseases	1.234 (0.835–1.824)	0.291
ADL	1.092 (1.027–1.160)	0.005
MNA scores	0.862 (0.772–0.963)	0.009

Model 1 and Model 2: Adjusted for age, smoke, exercise, number of chronic diseases, ADL

### The association of MNA score, anthropometric parameters and laboratory index with the occurrence of frailty

**Fig. 1:**
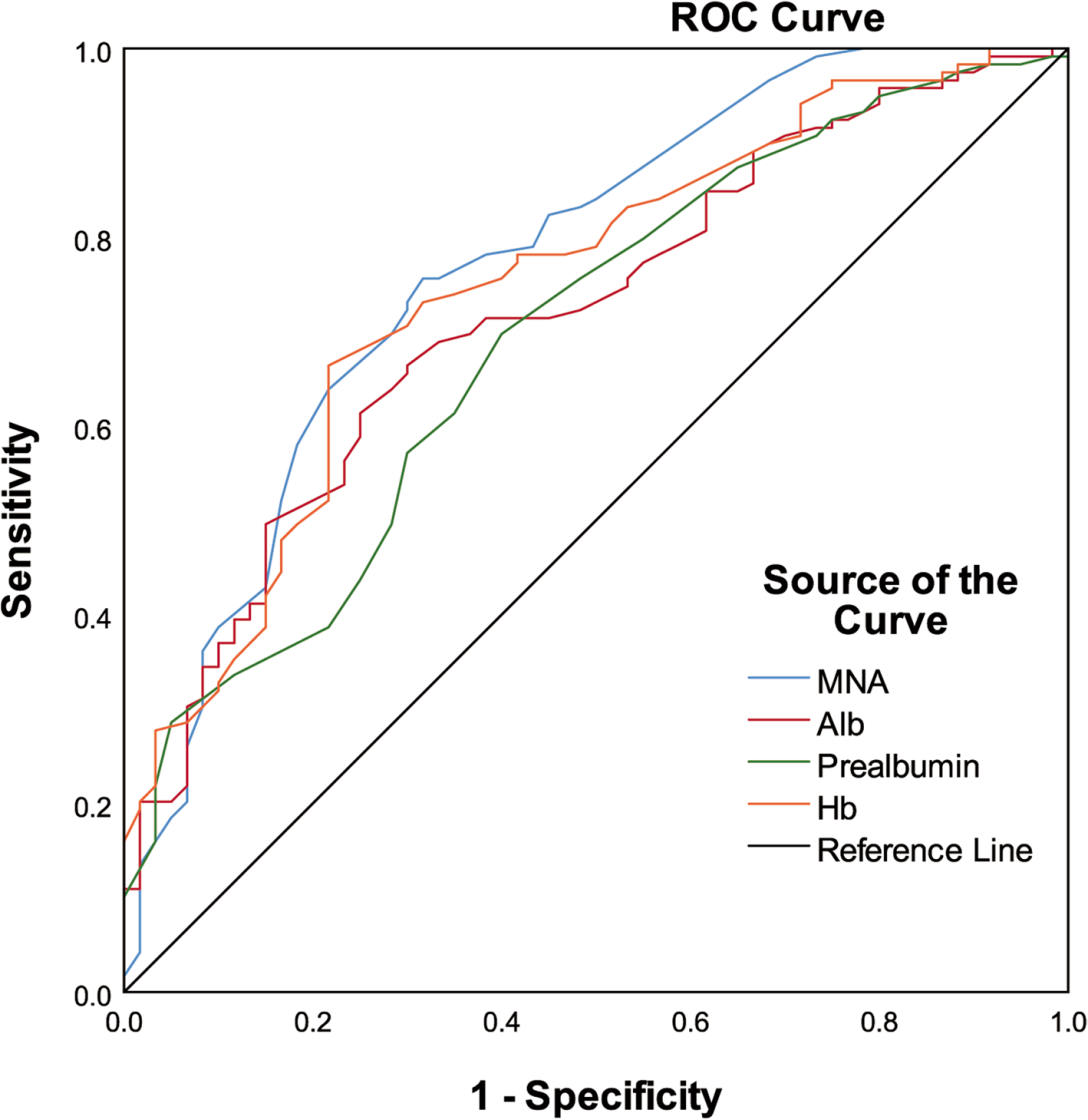
The receiver operating characteristic curve of different nutritional indexes for frailty

The correlation between different nutritional indexes on the occurrence of frailty in elderly was analyzed and compared. Results are shown in Table 4, the area under the curve of MNA score, albumin, prealbumin and hemoglobin was larger (AUC > 0.65), AUC was 0.773, 0.718, 0.693 and 0.743, respectively. However, through the comparison, it was found that MNA score did not exhibit obvious superiority for the occurrence of frailty than albumin, prealbumin and hemoglobin. The relevant ROC curve is shown in Fig. 1.

**Table 4:** Correlation between nutritional indexes and debilitation

	AUC (95% CI)	*P*	Cutoff value	Sensitivity (%)	Specificity (%)
MNA scores	0.773 (0.704–0.832)	< 0.001	20.6	75.63	68.33
BMI (kg/m2)	0.575 (0.499–0.648)	0.108	22.2	82.35	35.00
AC (cm)	0.621 (0.546–0.693)	0.009	25.3	78.99	43.33
CC (cm)	0.639 (0.564–0.709)	0.002	33.7	36.13	86.67
Hb (g/L)	0.748 (0.678–0.810)	< 0.001	134	66.39	78.33
Alb(g/L)	0.718 (0.646–0.783)	< 0.001	36.9	66.39	70.00
Prealbumin(g/L)	0.693 (0.620–0.760)	< 0.001	0.2	69.75	60.00
Cholesterol (mmmol/L)	0.632 (0.556–0.702)	0.003	4.46	57.98	68.33
Triglyceride (mmmol/L)	0.566 (0.490–0.640)	0.149	1.3	50.42	65.00
LDL-C (mmmol/L)	0.624 (0.548–0.695)	0.006	2.68	67.23	56.67

## Discussion

In addition to confirming previous reports, the advantage of our research lies in a more comprehensive analysis of the correlation between different nutritional indicators (anthropometric index, Mini Nutritional Assessment (MNA), laboratory related indicators) and frailty, and further study and explore the relationship between them by analyzing the receiver operating characteristic curve of different nutritional indexes. For a single nutritional index, our study found that albumin, prealbumin and hemoglobin are related to frailty, which provides a clue for screening frailty in the future. A review on frailty and malnutrition in 2016 supports this conclusion and points out that both malnutrition and frailty are reversible and the incorrectly identification can lead to the onset of disability. Nutritional therapy, protein rich diet, correction of vitamin D deficiency and physical activity are all basic aspects of multidisciplinary treatment of senile frailty [7]. A recent review has also shown the importance of quantitative (energy intake) and qualitative (nutritional quality) factors of nutrition in the development of senile frailty syndrome [17]. Nutritional research on the prevention and management of frailty shows that a healthier diet, such as the Mediterranean diet, is mainly to improve physical activity and walking speed and reduce the risk of falls [18].

Our study also found that there were significant differences in the proportion of smoking and exercise, the number of chronic diseases, the ADL score, MNA score and nutritional status between the frailty group and the non-frailty group (*P* < 0.05). Smoking is a risk factor for atherosclerosis [19], and atherosclerosis is a common mechanism of frailty [20]. So our study found that people who smoke for a long time were more prone to frailty. Myasthenia is a major component of the frailty syndrome, both are considered as a powerful predictor of incidence rate, disability and death in the elderly. Exercise intervention and nutritional supplements are the strategies for preventing and treating muscle wasting and frailty [21]. Chronic diseases are considered to be the main determinant of physical weakness [22]. According to the widely used construction of frailty phenotype proposed by Fried et al. [13], chronic diseases play a central role in the beginning or deterioration of frailty. This study also found that weakness is related to the number of chronic diseases [23]. Studies find that the decrease of hemoglobin [24–26] and albumin [26, 27] levels in circular blood is associated with the incidence of frailty, which also supports our study. In a recent systematic review, nutrition is identified as a means of delaying the occurrence of negative consequences of frailty in the elderly [28] and a method of slowing the development and progress of frailty in the age [17]. High quality diet matching is very important. Diet rich in antioxidants is an important factor in preventing and delaying the onset of frailty of old people [17]. Another study has found that there is a correlation between insufficient energy, nutrition intake and frailty of the elderly, energy intake ≤21 kcal / kg / day is associated with frailty, and intake of more than three nutrients (poor nutritional score) is significantly related to the frailty [29].

The occurrence of frailty may involve a variety of pathophysiological pathways. Age related changes (mitochondrial dysfunction, neuronal degeneration, cell senescence, stem cell degeneration, DNA methylation and injury, autophagy), gene and environment (cognition, nutrition, activity), energy metabolism changes caused by chronic diseases (cardiovascular disease, hypertension, diabetes, arthritis, obesity), stress response injury (chronic inflammation, HPA axis, autonomic nervous system), chronic inflammation, HPA axis, autonomic nervous system, decreased neural function, decrease of anabolic hormones, eventually leading to physical weakness [2]. The acceleration of low-grade inflammatory activity may be a potential cause of frailty syndrome. Studies have proved that the increased of cytokines IL6, CRP and TNF α levels are associated with frailty [30–32]. Changes in the endocrine system provide a basis for frailty. The endocrine hormones associated with frailty are testosterone and its precursor DHEA, parathyroid hormone (PTH), vitamin D and insulin-like growth factor [33]. Increased HbA1c levels have been repeatedly shown to be associated with a higher incidence rate of frailty syndrome [34, 35]. A series of commonly used serum markers have been evaluated as potentially associated with frailty, and glomerular filtration rate and albumin level are particularly noteworthy as potential indicators of frailty [33]. In addition to studying the relationship between different nutritional indicators and frailty, we also further study and explore the relationship between them by analyzing the receiver operating characteristic curve of different nutritional indexes. We found that albumin, prealbumin and hemoglobin were associated with frailty. It has also been found that elderly people at risk of malnutrition are more likely to develop geriatric syndrome (such as frailty syndrome or sarcopenia) than well nourished elderly people [36]. Therefore, early identification of malnutrition can reduce the incidence of senile frailty.

There are several limitations in current study. The main limitation is that this study is a cross-sectional design, so there is no statement of causality. Another limitation is the study samples are from one medical center, so the results are limited in universality. In conclusion, more prospective cohort studies in the elderly are needed to further understand the potential role of nutrition in preventing, delaying or reversing frailty syndrome.

## Conclusions

Our results suggest that malnutrition is closely related to frailty. As for single nutritional indexes, albumin, prealbumin and hemoglobin were found to be associated with frailty. Further cohort studies are needed to verify their ability to screen for frailty.

## Data Availability

The data and materials that support the findings of this study are available from the corresponding author upon reasonable request.

## Abbreviations

ADL: Ability of daily living
MNA: Mini Nutritional Assessment
AC: Arm circumference
CC: Calf circumference
Alb: Albumin
LDL-C: Low density lipoprotein cholesterol
